# Effects of high NH^+^_4_ on K^+^ uptake, culm mechanical strength and grain filling in wheat

**DOI:** 10.3389/fpls.2014.00703

**Published:** 2014-12-16

**Authors:** Lingan Kong, Mingze Sun, Fahong Wang, Jia Liu, Bo Feng, Jisheng Si, Bin Zhang, Shengdong Li, Huawei Li

**Affiliations:** Crop Research Institute, Shandong Academy of Agricultural SciencesJinan, Shandong, China

**Keywords:** ammonium (NH^+^_4_), culm mechanical strength, K^+^ flux, N remobilization, wheat (*Triticum aestivum* L.)

## Abstract

It is well established that a high external NH^+^_4_ concentration depresses many processes in plant development, but the underlying mechanisms are still not well understood. To determine whether the negative effects of high levels of NH^+^_4_ are related to competitive cation uptake, wheat was grown in a field with moderate (18 g N m^−2^) and high (30 g N m^−2^) supplies of NH^+^_4_ in the presence or absence of additional K^+^ (6 g K_2_O m^−2^) to examine culm mechanical strength, the main components of the vascular bundle, nitrogen (N) remobilization and the grain-filling rate. The results indicated that an excessive supply of NH^+^_4_ significantly decreased culm mechanical strength, the cellulose and lignin contents of vascular bundles, the N remobilization efficiency (NRE) and the grain-filling rate compared with a moderate level of NH^+^_4_. The additional provision of K^+^ considerably alleviated these negative effects of high NH^+^_4_, resulting in a 19.41–26.95% increase in culm mechanical strength during grain filling and a 34.59% increase in the NRE. An assay using the scanning ion-selective electrode technique (SIET) showed that the net rate of transmembrane K^+^ influx decreased by 84.62%, and measurements using flame photometry demonstrated that the K^+^ content decreased by 36.13% in wheat plants subjected to high NH^+^_4_. This study indicates that the effects of high NH^+^_4_ on culm mechanical strength, cellulose and lignin contents, the NRE and the grain-filling rate are probably associated with inhibition of K^+^ uptake in wheat.

## Introduction

Ammonium (NH^+^_4_) is an important source of inorganic N and can be used by plants as the sole N source. However, high levels of ambient NH^+^_4_ can be toxic to plant growth, resulting in many symptoms, including stunted root growth, yield reduction and leaf chlorosis (Britto and Kronzucker, 2002; Balkos et al., 2010; Li et al., 2010; Ariz et al., 2011; Chen et al., 2013). In rice (*Oryza sativa* L.), the anatomical traits of the culm are altered when high N is provided, leading to a reduction in culm mechanical strength and an increase in lodging scores (Yang et al., 2009). When larger quantities of urea (transformed to NH^+^_4_ through urease hydrolysis) are supplied, the efficiency of silicon in imparting rigidity in rice plants at low N doses is greatly reduced (Idris et al., 1975). In cereal crop production, lodging resulting from low mechanical strength severely damages the vascular bundles (Kashiwagi et al., 2008), thereby affecting the transport of water, nutrients and reserves contained in vegetative organs to the developing grain and decreasing grain yield and quality. Little information is currently available concerning the effects of NH^+^_4_ on culm mechanical strength and reserve transport in wheat. However, field observations and experience show that when wheat is grown at a high N rate, there appears to be an increased occurrence of lodging compared with growth at a moderate N rate, indicating that culm strength is low under high N levels. In *Arabidopsis thaliana*, when excessive N is supplied, the N concentration in the biomass increases significantly, whereas the N remobilization efficiency (NRE) decreases compared with moderate N application (Masclaux-Daubresse and Chardon, 2011). In a previous study, we found that application of excessive urea decreases the export of flag leaf-stored protein to the developing grains (Kong et al., 2012). Therefore, improving the NRE is a good strategy for achieving a high grain yield and crop quality under high N conditions.

The majority of studies investigating the toxicity of NH^+^_4_ at high concentrations have been associated with NH^+^_4_ assimilation and ion imbalances due to the decreased uptake of essential cations, such as K^+^, Mg^2+^ and Ca^2+^ (Barker et al., 1967; Roosta and Schjoerring, 2008; ten Hoopen et al., 2010), and they are often associated with the availability of K^+^ in particular (Yang et al., 2009). Because NH^+^_4_ can be transported through plant K^+^ transporters and channels, NH^+^_4_ toxicity may be attributed to unregulated NH^+^_4_ uptake via these transporters, especially at low K^+^ levels (ten Hoopen et al., 2010). It was recently proposed that NH^+^_4_ toxicity in NH^+^_4_-fed plants originates from NH_3_ uptake by plants through one component of the low-affinity transport system (LATS) for NH^+^_4_ and from interference with K^+^ transport through the second component (Ariz et al., 2011). In *Arabidopsis*, stimulation of the NH^+^_4_ efflux in the elongation zone following treatment with elevated NH^+^_4_ is linked to root growth inhibition by NH^+^_4_ (Li et al., 2010). The NH^+^_4_ efflux significantly enhances futile and energy-costly NH^+^_4_ cycling at the plasma membrane in rice (Chen et al., 2013). Although a variety of hypotheses have been proposed to explain the mechanisms underlying NH^+^_4_ toxicity, no single convincing mechanism has yet been able to fully account for this toxicity (Roosta and Schjoerring, 2008; Chen et al., 2013).

Under field conditions, we have often observed the adverse effects of high NH^+^_4_ provision on wheat. Therefore, in this study, experiments were conducted in both the field and laboratory to examine the cellulose and lignin contents of the vascular bundle, culm mechanical strength and the NRE under treatment with moderate or high NH^+^_4_, or with high NH^+^_4_ combined with an elevated K^+^ supply. The main objectives of this study were to investigate the effects of excessive external NH^+^_4_ on wheat growth and to determine whether elevated K^+^ concentrations can alleviate these adverse effects of high NH^+^_4_.

## Materials and methods

### Plant materials

A field experiment was conducted at an experimental station (36°42′ N, 117°4′ E; altitude 48 m) of the Shandong Academy of Agricultural Sciences, China. The climate in this region is continental and warm, with an average annual temperature of 13.6°C and an average rainfall of ~600 mm. The soil type was classified as sandy loam, with a pH of 7.2. The top 40 cm of the soil contained 2.13% organic matter, 66.2 mg kg^−1^ hydrolysable nitrogen, 25.3 mg kg^−1^ rapidly available phosphorous and 152.4 mg kg^−1^ rapidly available potassium.

The winter wheat (*Triticum aestivum* L.) variety Jimai 22, developed by the Crop Research Institute of the Shandong Academy of Agricultural Sciences, Jinan, was used in the experiment and was sown on October 8, 2012, at a rate of 375 grains per m^2^. The experiments were laid out in a split-plot design with three treatments and four replications. The treatments included moderate NH^+^_4_ (18 g N m^−2^), high NH^+^_4_ (30 g N m^−2^) and high NH^+^_4_ plus additional K^+^ (K^+^_add_; 6 g K_2_O m^−2^). At sowing, 5 g N m^−2^, 9 g P_2_O_5_ m^−2^, and 9 g K_2_O m^−2^ were applied per treatment as basal nutrition. At the first node stage (the end of tillering), 13 g N m^−2^, 25 g N m^−2^, or 25 g N m^−2^ plus 6 g K_2_O m^−2^ was top dressed in all three treatments, followed immediately by irrigation. N was supplied as NH_4_Cl. The field-grown plants were used for data collection unless otherwise stated.

### Measurement of culm mechanical strength

The culm mechanical strength of the middle point of the basal second internode without a leaf sheath was measured using a handmade device. The device contains two semicircular semicircular grooves (5 cm apart) with approximately the same diameter as the wheat culm. During measurement, the second internode was set on the grooves, and a pallet was hung at the center of the internode; fine sand was then gradually added to the pallet until the stem broke. The total weight of the added sand and the pallet was subsequently determined using a balance. Culm mechanical strength was directly expressed as the weight required to break the internode.

### Histochemistry

For histochemical localization of lignin, Wiesner reactions were performed using the method of Speer (1987). Briefly, transverse sections of the wheat culm second internode were cut freehand with a razor blade. Fresh sections were then incubated for 3 min in a 2% phloroglucinol (w/v), 95% EtOH solution, followed by 3 min of incubation in 50% HCl and subsequent mounting in 50% glycerol, in which phloroglucinol produces a red-pink product under acidic conditions, primarily through reaction with lignin cinnamaldehyde groups. The plant sections were examined directly under a light microscope, and digital images were recorded using an AxioCam MRC camera (Zeiss Axioskop 40, Leica, Germany). The optical density of stained lignin was quantified using the Image-Pro Plus 6.0 software (Media Cybernetics, Silver Springs, MD) and expressed on a scale of 0–2.

For cellulose staining, second internodes were transverse sectioned and stained freehand with a 0.005% (w/v) solution of Fluorescent Brightener 28 (FB 28, Calcofluor White M2R; Sigma) for 5–10 min. The stained sections were observed under a fluorescence microscope (Zeiss Axioskop 40) using a BP 365 excitation filter, an FT 395 chromatic beam splitter and an LP 420 barrier filter. Digital images were recorded using an AxioCam MRC camera. The intensity of fluorescence was quantified with the Image-Pro Plus 6.0 software, and the cellulose content was expressed as the exponent optical density, ranging from 0 to 2.

### Fourier transform infrared (FTIR) spectroscopy

Freehand sections (*c*. 40 μm) were oven-dried at 60°C, and the vascular bundles were removed using a razor blade under an anatomical microscope before FTIR analysis. The FTIR spectra were recorded using an FTIR spectrometer (Magna-IR 750, Thermo Nicolet, Kanagawa, Japan) equipped with a Mercury–Cadmium–Telluride detector. The spectra of each sample were obtained in the range of 4000–400 cm^−1^ at a resolution of 4 cm^−1^ with 128 co-added interferograms and were normalized to obtain the relative absorbance.

### Determination of shoot N contents

Entire wheat shoots were dried at 70°C to constant weight and ground to pass through a 1-mm sieve. The samples (1 g of dry weight) were then placed in a Kjeldahl flask, and 20 ml of concentrated H_2_SO_4_ was added. After digestion, the solutions were cooled and diluted with deionized water to the specified volume. The total shoot N content was determined using the standard Kjeldahl procedure (Watkins et al., 1987). N remobilization is defined as the difference in the amount of shoot N between anthesis and the harvest stage. The NRE was calculated as the ratio between remobilized N and the total shoot N content at anthesis.

### Grain filling

Based on grain development, five growth stages were designated for measurement: 0 days after anthesis (DAA) (end of anthesis); 8 DAA (milk development stage); 16 DAA (soft dough development stage); 24 DAA (hard dough development stage); and 32 DAA (ripening stage). The ears were collected at each stage and dried at 70°C for 48 h to a constant mass. The samples were then manually threshed, and the grains were weighed. The data were averaged from three replicates, each with 60 ears.

### Measurement of K^+^ contents

Fifteen germinated wheat seeds were transplanted into plastic basins (25 cm high and 20 cm in diameter) containing sterilized wet sand. The plants were then grown at 20–22°C under an 18-h photoperiod (white fluorescent light; 400 μmol m^−2^ s^−1^). The plants were watered daily with full-strength Hoagland's nutrient solution (HNS) as a control; with HNS supplemented with 10 mM NH_4_Cl as the high NH^+^_4_ treatment; or with HNS supplemented with 10 mM NH_4_Cl and 6 mM KCl as the high NH^+^_4_ plus additional K^+^ treatment. For the K^+^ analysis, entire 40-day-old wheat plants were collected, washed with DD H_2_O and oven-dried at 70°C for 48 h. The dried material was finely powdered and then subjected to wet digestion with HNO_3_:HClO_4_ (4:1) under shaking for 20 min at 200 rpm. The samples were filtered through Whatman No. 2 filter paper. The resulting solutions were appropriately diluted, and the K^+^ content was measured using a flame photometer (FP640, Shanghai, China).

### K^+^ flux analysis using the scanning ion-selective electrode technique (SIET)

For SIET, wheat seeds were surface sterilized in an aqueous solution of 1% NaClO for 5 min. The seeds were then washed several times with sterilized water, placed on wet filter paper in Petri dishes and incubated in distilled water. After germination, the seeds were transferred to larger plastic vessels and cultured hydroponically in full-strength HNS. The net K^+^ fluxes into the root epidermal cells were measured noninvasively in 15-day-old seedlings using SIET (BIO-001A SIET system; Younger USA Sci. & Tech. Corp., Amherst, MA, USA; Applicable Electronics Inc., Forestdale, MA, USA and ScienceWares Inc., East Falmouth, MA, USA). Recordings of steady-state K^+^ fluxes were performed as described by Sun et al. (2009). Prior to the measurements, the probes were calibrated in a solution (0.05 mM NH_4_NO_3_, 0.05 mM KCl, 0.1 mM CaCl_2_, and 0.3 mM MES, pH 6.0) for 10 min. The steady fluxes were assayed in measuring solution (0.1 mM NH_4_NO_3_, 0.1 mM KCl, 0.1 mM CaCl_2_, and 0.3 mM MES, pH 6.0) containing 10 mM NH_4_Cl for approximately 10 min to verify that a steady-state condition was reached. Then, the transient K^+^ kinetics in the root epidermal cells 14 mm from the apex were measured for an additional 30 min (**Figures 6A,B**). As a control, the K^+^ flux was measured in measuring solution not containing 10 mM NH_4_Cl.

### Statistical analysis

All of the data were subjected to analysis of variance (ANOVA) using the Data Processing System (DPS) statistical software (v.14.10, Refine Information Tech. Co., Ltd., Hangzhou, Zhejiang, China) (Tang and Zhang, 2013). The data are presented as the mean ± standard deviation. The treatment means were compared using the least significant difference (LSD) test at *P* < 0.05.

## Results

### Decrease in culm mechanical strength due to high NH^+^_4_

**Figure 1:**
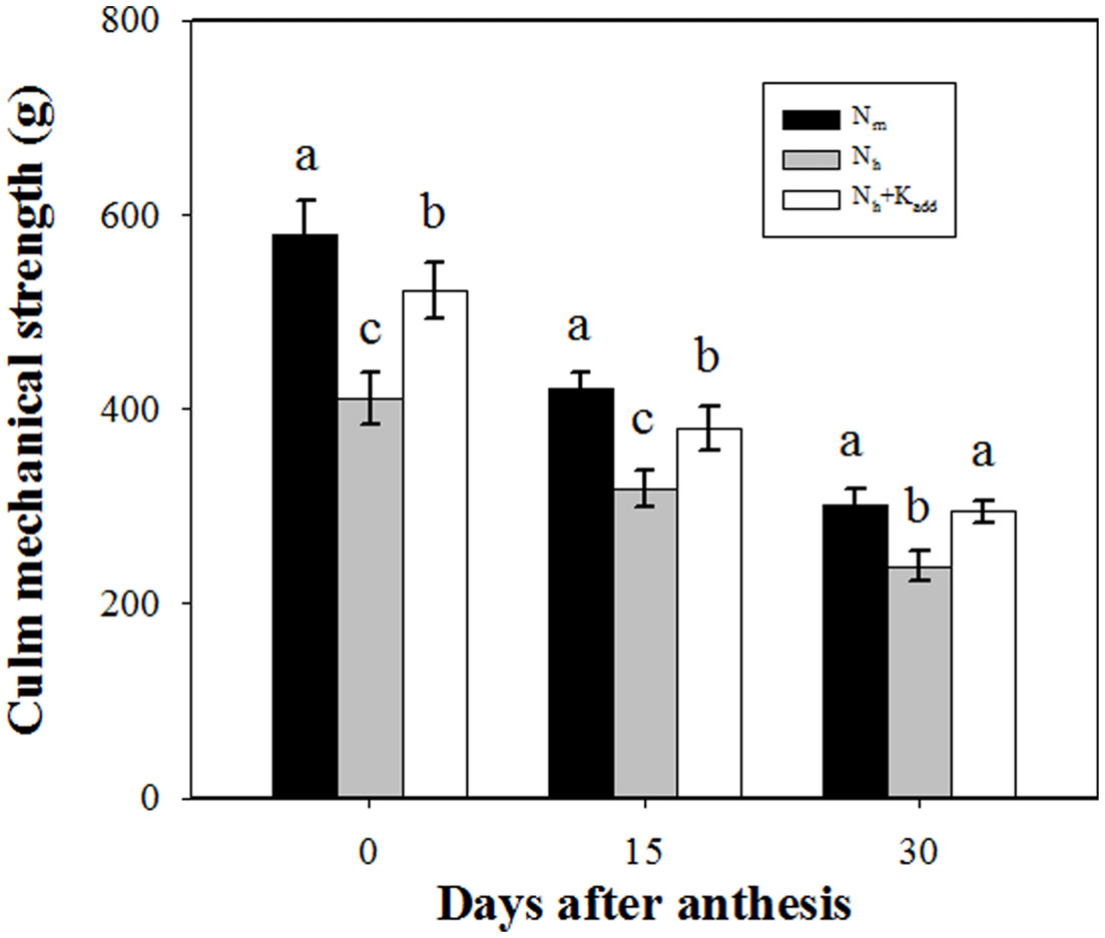
**Effects of moderate NH^+^_4_ (18 g N m^−2^; N_m_), high NH^+^_4_ (30 g N m^−2^; N_h_) and high NH^+^_4_ plus additional K^+^ (6 g K_2_O m^−2^; N_h_ + K^+^_add_) on the culm mechanical strength of the basal second internode in wheat**. The columns labeled with different letters differed significantly at *P* < 0.05. The bars represent the standard deviation, *n* = 30.

Because lodging often occurred from the base of the plants, we determined the culm mechanical strength of the basal second internode of the wheat plants. Figure 1 shows that under field conditions, culm mechanical strength decreased continuously from anthesis to ripening. The application of high NH^+^_4_ (30 g N m^−2^) led to significantly lower culm mechanical strengths, which were reduced by 29.20, 26.37, and 20.88% at 0, 15 and 30 DAA, respectively, compared with the treatment with a moderate level of NH^+^_4_ (18 g N m^−2^). Under high NH^+^_4_ conditions, K^+^_add_ improved culm mechanical strength by 26.95, 19.41, and 23.46% at 0, 15 and 30 DAA, respectively, compared with the treatment without K^+^_add_. A decrease in culm mechanical strength under high NH^+^_4_ (HNS containing 10 mM NH^+^_4_) (Figure S1B) and an improvement in the K^+^_add_ treatment (Figure S1C) were also observed in wheat plants in a sand culture system. However, the adverse effect of high NH^+^_4_ was only partially reversed by elevated K^+^.

### Differential effects of high NH^+^_4_ and K^+^_add_ on cellulose and lignin contents

**Figure 2:**
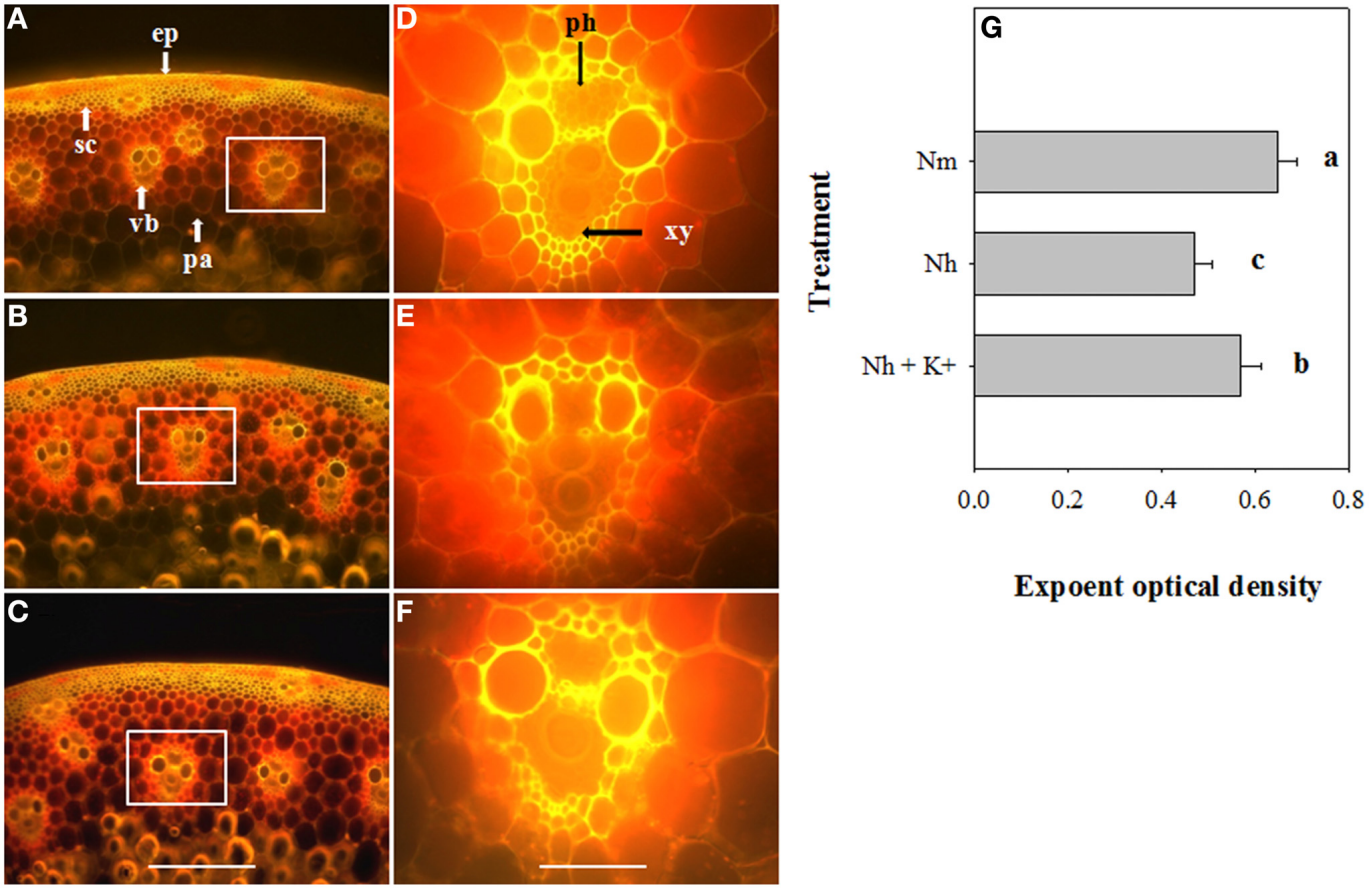
**Microscopy images of Calcofluor staining of cellulose in sections of the second internode of wheat plants at 16 DAA, showing that the maximum cellulose content was mainly present in the vascular bundles and sclerenchyma**. The relative differences in the cellulose contents of wheat under moderate NH^+^_4_ (18 g N m^−2^) **(A)**, high NH^+^_4_ (30 g N m^−2^) **(B)** and high NH^+^_4_ combined with K^+^_add_ (6 g K_2_O m^−2^) **(C)** are shown. **(D)**, **(E)**, and **(F)**: higher magnification images of the areas highlighted in **(A)**, **(B)**, and **(C)**, respectively. The presence of cellulose is indicated by luminous yellow coloration. The images represent at least 15 cross-sections from different plants for each treatment. **(G)**: Effects of moderate NH^+^_4_, high NH^+^_4_ and high NH^+^_4_ combined with K^+^_add_ on cellulose deposition in the cell wall of the vascular bundles. The fluorescence intensity was quantified using Image-pro Plus 6.0 software. The columns labeled with different letters differed significantly at *P* < 0.05. ep, epidermis; pa, parenchyma cells; ph, phloem; sc, sclerenchyma cells; vb, vascular bundle; xy, xylem. Bars **(A–C)** 200 μm; **(D–F)** 800 μm.

**Figure 3:**
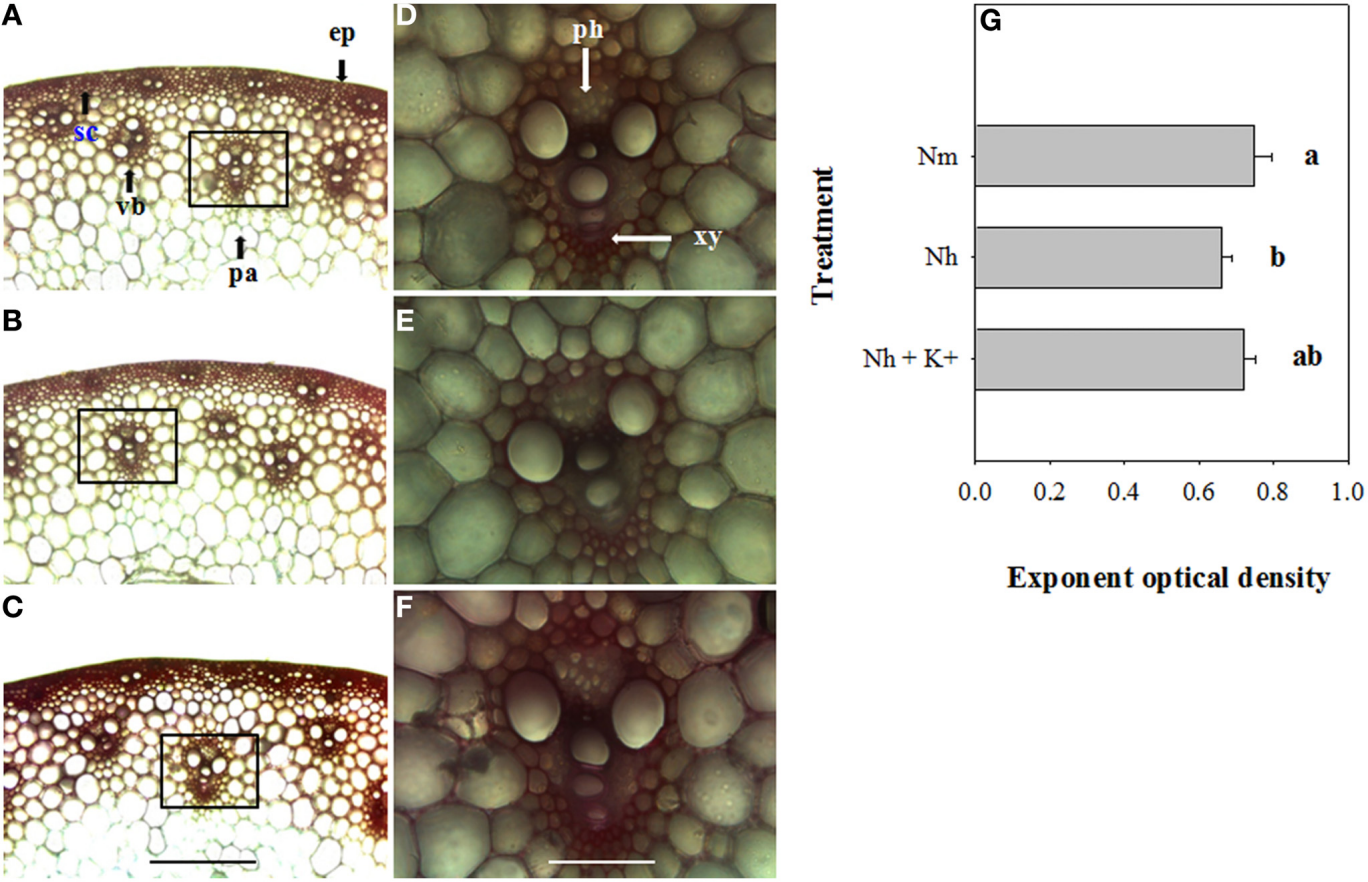
**Microscopic images of the Wiesner staining of lignin in sections of the second internode of wheat plants at 16 DAA, showing that the maximum lignin content was mainly present in the vascular bundles and sclerenchyma, as indicated by red-pink coloration**. The relative differences in the lignin content under moderate NH^+^_4_ (18 g N m^−2^) **(A)**, high NH^+^_4_ (30 g N m^−2^) **(B)** and high NH^+^_4_ combined with K^+^_add_ (6 g K_2_O m^−2^) **(C)** are shown. **(D–F)**: higher magnification images of the areas highlighted in **(A–C)**, respectively. The presence of lignin is indicated by red-pink coloration. The images are representative of at least 15 cross-sections from different plants for each treatment. **(G)**: Effects of moderate NH^+^_4_, high NH^+^_4_ and high NH^+^_4_ combined with K^+^_add_ on lignin deposition in the cell wall of the vascular bundles. The optical intensity was quantified using the Image-pro Plus 6.0 software. The columns labeled with different letters differed significantly at *P* < 0.05. ep, epidermis; pa, parenchyma cells; ph, phloem; sc, sclerenchyma cells; vb, vascular bundle; xy, xylem. Bars **(A–C)** 200 μm; **(D–F)** 800 μm.

To determine whether high NH^+^_4_ affects the contents of lignin and cellulose and whether elevated K^+^ modifies their biosynthesis and localization, culm cross-sections from the different treatments were stained with Calcofluor, to visualize cellulose, or with Wiesner reagents, to visualize lignin (Figures 2, 3). As shown in the obtained images and through analysis with Image-pro Plus 6.0 software, the cellulose fluorescence intensity, particularly in the vascular bundles, was weaker in high-NH^+^_4_-treated wheat (Figures 2B,E,G) than in moderate-NH^+^_4_-treated wheat (Figures 2A,D,G). When the wheat plants were exposed to high NH^+^_4_, K^+^_add_ significantly promoted cellulose deposition in the vascular bundles (Figures 2C,F,G). Similarly, the amount of lignin decreased in the internodes of the wheat plants under high NH^+^_4_, as indicated by the weaker red-pink color observed (Figures 3B,E,G) and the measurements of optical density performed using the Image-Pro Plus 6.0 software, compared with the moderate NH^+^_4_ treatment (Figures 3A,D,G). Additionally, K^+^_add_ significantly relieved the reduction of the lignin content detected under high NH^+^_4_ (Figures 3C,F,G).

**Figure 5A** illustrates the typical FTIR spectra obtained from the vascular bundles of the basal second internode of wheat plants at 16 DAA; the data point shown in each spectrum is an average of four measurements. In all three treatments, peaks occurred at wavelengths of approximately 2920 and 2850 cm^−1^, which were ascribed to the aliphatic saturated C-H stretching vibration that takes place in polysaccharides, particularly cellulose and hemicelluloses (Lichtfouse et al., 1998; Kaushik et al., 2010). A prominent peak at 1248 cm^−1^ and a shoulder peak at 1130 cm^−1^ represent the C-H, O-H, or CH_2_ bending frequencies (Himmelsbach et al., 2002) and are indicators of lignin (Kaparaju and Felby, 2010). An indistinct band at 897 cm^−1^ indicates the typical structure of cellulose (due to the β-glycosidic linkages of the glucose ring of cellulose) (Gañán et al., 2004).

To quantitatively distinguish the changes in the cellulose and lignin contents of the vascular bundles, difference spectra were generated through digital subtraction of the spectra of wheat plants treated with high levels of NH^+^_4_ from those of plants treated with moderate NH^+^_4_ or through digital subtraction of the spectra of K^+^_add_ treatment from those without additional K^+^ under high NH^+^_4_. In the different spectra recorded, distinct peaks appeared at 2920, 2850, 1248, 1130, and 897 cm^−1^. The decreased absorbance intensity due to a high level of NH^+^_4_ indicated that the cellulose and lignin contents decreased, whereas the positive values due to elevated K^+^ treatment under high NH^+^_4_ conditions suggested that the cellulose and lignin contents increased in the vascular bundles of wheat.

### Changes in shoot N concentrations

As expected, the total shoot N concentration (%) in wheat during grain filling was consistently higher in the excessive compared with the insufficient NH^+^_4_ treatment, suggesting that high NH^+^_4_ promoted N uptake by the plants (Table 1). At 0 DAA, the greater total shoot N concentration caused by a high NH^+^_4_ supply was further increased by the K^+^_add_ treatment. The shoot N concentration gradually decreased during the grain-filling period in all three treatments. However, the shoot N concentration decreased more sharply under moderate NH^+^_4_ than at high NH^+^_4_; as a result, high NH^+^_4_ led to a lower NRE compared with the moderate NH^+^_4_ treatment. Under high NH^+^_4_ conditions, the shoot N concentration was greater from 0 to 16 DAA in the K^+^_add_ treatment than in the treatment without additional K^+^, but the concentrations in these treatments were similar at maturity (32 DAA); consequently, the K^+^_add_ treatment resulted in a greater NRE. These data strongly indicate that the K^+^_add_ treatment might increase the N-use efficiency under high NH^+^_4_ conditions.

**Table 1:** **Effects of NH^+^_4_ and K^+^ on the total shoot N concentration (%) and NRE (%) in wheat**.

**NH^+^_4_/K^+^ rate (g m^−2^)**	**Total shoot N concentration (%)**			**NRE^b^ (%)**
	**0 DAA^a^**	**16 DAA**	**32 DAA**	
N_m_ (18)	1.08 ± 0.05c^3^	0.84 ± 0.09b	0.62 ± 0.05b	42.53 ± 3.30a
N_h_ (30)	1.24 ± 0.07b	0.98 ± 0.08ab	0.96 ± 0.06a	22.35 ± 2.64c
N_h_ (30) + K^+^_add_ (6 g K_2_O kg)	1.40 ± 0.07a	1.05 ± 0.08a	0.98 ± 0.04a	30.08 ± 2.31b

^a^DAA, days after anthesis.

^b^NRE, N remobilization efficiency.

^c^The values represent the means and standard deviations of four replicates. Means followed by the same letters within a column are not significantly different by Tukey's test (P < 0.05).

### Grain-filling rate

The grain dry mass decreased when the wheat plants were treated with high NH^+^_4_, especially during the early stages of grain filling (8, 16, and 24 DAA), compared with the application of moderate NH^+^_4_. Under high NH^+^_4_ conditions, additional K^+^ increased the grain dry mass throughout the grain-filling period (Table 2); i.e., the elevated K^+^ supply increased the grain-filling rate when wheat was subjected to high NH^+^_4_.

**Table 2:** **Effects of NH^+^_4_ and K^+^ on grain dry mass (mg ear^−1^) during grain filling**.

**NH^+^_4_/K^+^ rate (g m^−2^)**	**Developmental stage (DAA^a^)**				
	**0**	**8**	**16**	**24**	**32**
N_m_ (18)	0	0.28 ± 0.02a^2^	0.64 ± 0.02a	1.06 ± 0.07a	1.28 ± 0.06ab
N_h_ (30)	0	0.24 ± 0.02b	0.59 ± 0.02b	1.03 ± 0.04a	1.25 ± 0.03b
N_h_ (30) + K^+^_add_ (6 g K_2_O kg^−1^)	0	0.27 ± 0.01ab	0.63 ± 0.03ab	1.11 ± 0.07a	1.36 ± 0.07a

^a^DAA, days after anthesis.

^b^The values represent the means and standard deviations of four replicates. Means followed by the same letters within a column are not significantly different by Tukey's test (P < 0.05).

### Inhibition of K^+^ uptake under high NH^+^_4_

A sand culture experiment was conducted to evaluate K^+^ uptake by the wheat plants using flame photometry. The high NH^+^_4_ treatment significantly decreased the K^+^ concentration in whole wheat plants compared with the moderate NH^+^_4_ supply (*P* < 0.01). As expected, under high NH^+^_4_ conditions, the K^+^_add_ treatment increased the K^+^ content and improved culm mechanical strength (Figure 1). However, K^+^_add_ only partly relieved the significant reduction in K^+^ contents caused by high NH^+^_4_ (**Figure 5**).

### Root K^+^ fluxes in response to high NH^+^_4_

To further evaluate whether high NH^+^_4_ influences K^+^ uptake in wheat seedlings, we measured the net K^+^ flux responses in root epidermal cells using SIET (**Figure 6A**). In the roots of wheat plants that were not treated with 10 mM NH_4_Cl, the net K^+^ influx into the root epidermal cells was determined (**Figure 6B**). SIET analyses showed that the average influx rate was approximately 17.88 pmol cm^−2^ s^−1^ (**Figure 6C**). However, adding 10 mM NH_4_Cl to the measuring solution resulted in a remarkable decrease in the K^+^ influx rate, which dropped to an average of 2.75 pmol cm^−2^ s^−1^ (**Figure 6C**).

## Discussion

Previous studies have shown that high N application leads to thinner stems and significant increases in lodging, the lodging angle and the lodging score in wheat (Tripathi et al., 2003) and rice (Yang et al., 2009). Under a high N level (240 kg ha^−1^, supplied as urea), the culm and root strengths of wheat were found to be 20% and 17% weaker, respectively, compared with the application of 160 kg N ha^−1^ (Crook and Ennos, 1995), suggesting that high N application led to a lower culm strength and an increased occurrence of lodging. However, these investigations did not provide information explaining the mechanisms underlying these results. Because the basal part of the culm plays an important role in lodging resistance, as it provides a lever to hold the plant upright (Neenan and Spencer-Smith, 1975), and because culm breakage usually occurs at lower internodes (Kashiwagi et al., 2008), we determined the culm mechanical strength of the basal second internode. The results showed that the application of high NH^+^_4_ significantly decreased culm mechanical strength.

Nitrogen and potassium are two macroelements that are essential for plant growth. Although plants exhibit a wide variety of transport systems for the acquisition of these elements, competition for uptake between plants still exists and has become one of the main topics of studies conducted by biologists and agronomists. Rice growth is negatively affected by elevated NH^+^_4_, particularly under low K^+^ levels, and NH^+^_4_ toxicity could be relieved by elevated K^+^ (Balkos et al., 2010). Similar findings have been reported in *Arabidopsis* (Li et al., 2010). Therefore, we expected that elevated K^+^ could compensate for the reduction in culm mechanical strength observed under high NH^+^_4_ conditions. Indeed, in this study, we found that K^+^_add_ alleviated the negative effects of high NH^+^_4_ on culm strength (Figure 1 and Figure S1). The data provided herein suggest that the plant K^+^ status might be involved in the high NH^+^_4_-induced reduction in culm mechanical strength. These results are highly consistent with those of previous studies. In a field trial with maize (*Zea mays* L.), the application of K^+^ was shown to significantly affect stem strength and stalk breakage (Melis and Farina, 1984). In rice, the K^+^ culm content is closely correlated with culm mechanical strength because proper K^+^ nutrition is associated with the lignification of sclerenchyma cells and vascular bundles, thereby strengthening the culms and increasing lodging resistance (De Datta and Mikkelsen, 1985; Zhang et al., 2010). To date, there is no any direct evidence to support that K^+^ strengthens cellulose and lignin deposition. However, it is widely accepted that K^+^ play a key role in photosynthesis and metabolism of the resulting carbohydrates in plants (White and Karley, 2010; Hafsi et al., 2014). Considering that both cellulose and lignin are carbohydrates or it's derivate and several pieces of indirect evidence presented herein are supportive, we could postulate that K^+^ is involved in the cellulose and lignin deposition and thus in culm mechanical strength.

**Figure 4:**
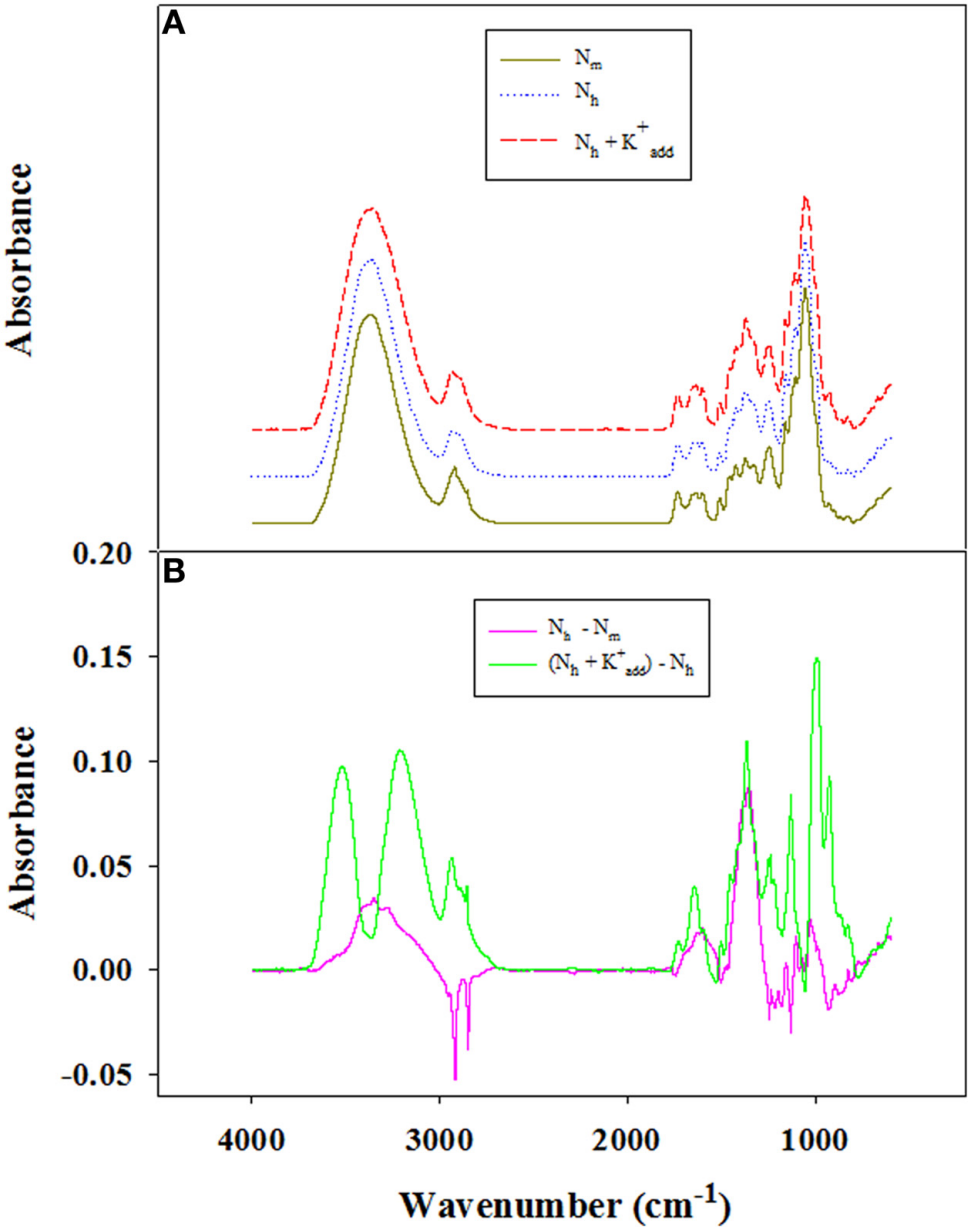
**FTIR spectra of vascular bundles separated from transverse sections of the second internodes of wheat plants at 16 DAA. (A)** FTIR spectra obtained from the vascular bundles of wheat under moderate NH^+^_4_ (18 g N m^−2^; N_m_), high NH^+^_4_ (30 g N m^−2^; N_h_) and high NH^+^_4_ combined with K^+^_add_ (6 g K_2_O m^−2^; N_h_ + K^+^_add_). The spectra are separated for comparison. **(B)** Different spectra generated through digital subtraction of the spectrum of the vascular bundles of wheat treated with high NH^+^_4_ from that of wheat treated with moderate NH^+^_4_ (N_h_-N_m_), or subtraction of the spectrum of vascular bundles of wheat treated with high NH^+^_4_ and K^+^_add_ from that of wheat treated with high NH^+^_4_ without an additional K^+^ supply [(N_h_ + K^+^_add_) −N_m_].

Mechanical strength is largely dependent on the chemical and biochemical components of the cell wall (Kashiwagi and Ishimaru, 2004; Kashiwagi et al., 2008). Generally, lignin and cellulose, which are the main biochemical components of plant tissues, particularly in the vascular bundles, are closely associated with culm mechanical strength (Yang et al., 2009). Cellulose usually constitutes 20–30% or 40–90% of the dry weight of primary or secondary walls, respectively, varying with the cell type (Taylor et al., 1999). Moreover, lignin can be incorporated into the cell wall to enhance its mechanical strength. In the rice mutant *brittleculm1* (*bc1*), altered biosynthesis of cellulose, hemicellulose and lignin in the culms reduces secondary cell wall thickness and mechanical strength (Li et al., 2003). In wheat, the expression of *COMT*, a gene involved in lignin biosynthesis in the developing culm, is associated with culm rigidity and lodging traits (Ma et al., 2002). The Ta*CAD1* gene is also responsible for lignin synthesis, and the roles of lignin in maintaining stem strength and lodging resistance were further confirmed in maize (Halpin et al., 1998) and sorghum (*Sorghum vulgare* Pers.) (Sattler et al., 2009) using *CAD* mutants. In the present study, through FTIR, histochemistry and Image-Pro Plus software analyses, we found that the application of high NH^+^_4_ decreased the cellulose and lignin contents in the vascular bundles of the second internode (Figures 2–4). In maize, K^+^ stimulates rapid expression of phenylalanine ammonia-lyase and enhances the activities of tyrosine ammonia-lyase, cinnamyl alcohol dehydrogenase and phenoloxidase, thereby increasing lignin biosynthesis (Liu et al., 2007). Considering that culm strength is correlated with the contents of cellulose and lignin and, more importantly, with K^+^ status, we speculate that an elevated K^+^ supply likely alleviates the negative effect of NH^+^_4_ on the deposition of cellulose and lignin in vascular bundles. Indeed, using FTIR and histochemistry, we observed that the K^+^_add_ treatment increased the contents of both of these cell wall components (Figures 2–4).

It is widely known that as major components, cellulose and lignin are mainly deposited in the walls of certain specialized cells, such as the tracheary elements, sclerenchyma and phloem fibers (also shown in the present study; Figures 2, 3). Considering that cellulose and lignin impart rigidity and structural support to the wall and strongly assist in the transport of water and nutrients within xylem tissue by decreasing the permeability of the cell wall (Ma et al., 2002), we decided to investigate the transport of shoot reserves to developing organs. In this study, we found that high NH^+^_4_ nutrition influenced N transport from vegetative organs to the developing grains (Table 1) and decreased the grain-filling rate (Table 2). Given that carbohydrates account for the majority of the wheat grain composition and that more significant differences in the grain-filling rate than in the NRE are observed between different treatments (Tables 1, 2), the decrease in the grain-filling rate may suggest that the nutrient translocation efficiency is not only determined by the lower permeability of the cell wall, but by the tissue C/N balance as well. Interestingly, elevated K^+^ also alleviates the negative effects of high NH^+^_4_ on N remobilization and grain filling. These findings are consistent with previous studies in *Arabidopsis thaliana* in which the NRE was shown to be greater under low N than under a high N supply (Masclaux-Daubresse and Chardon, 2011), and the toxicity of high NH^+^_4_ can be alleviated by K^+^ supplementation (Cao et al., 1993).

NH^+^_4_ nutrition dramatically affects cation uptake by plants, leading to a reduction of cation contents (Szczerba et al., 2006, 2008). The mechanism underlying this effect is unknown, but it is commonly considered to result from direct competition between NH^+^_4_ and other cations for the transmembrane through common pathways (ten Hoopen et al., 2010). In particular, K^+^ and NH^+^_4_ may use the same channels because these cations are highly similar regarding their charge, size and hydration energy, which are characteristics that are important for membrane transport (Wang et al., 1996; White, 1996; Szczerba et al., 2008). K^+^ channels are an important component of the LATS for NH^+^_4_ (ten Hoopen et al., 2010). Therefore, it has been speculated that these negative effects of high NH^+^_4_ on wheat might be related to the modification of K^+^ flux in root cells.

**Figure 5:**
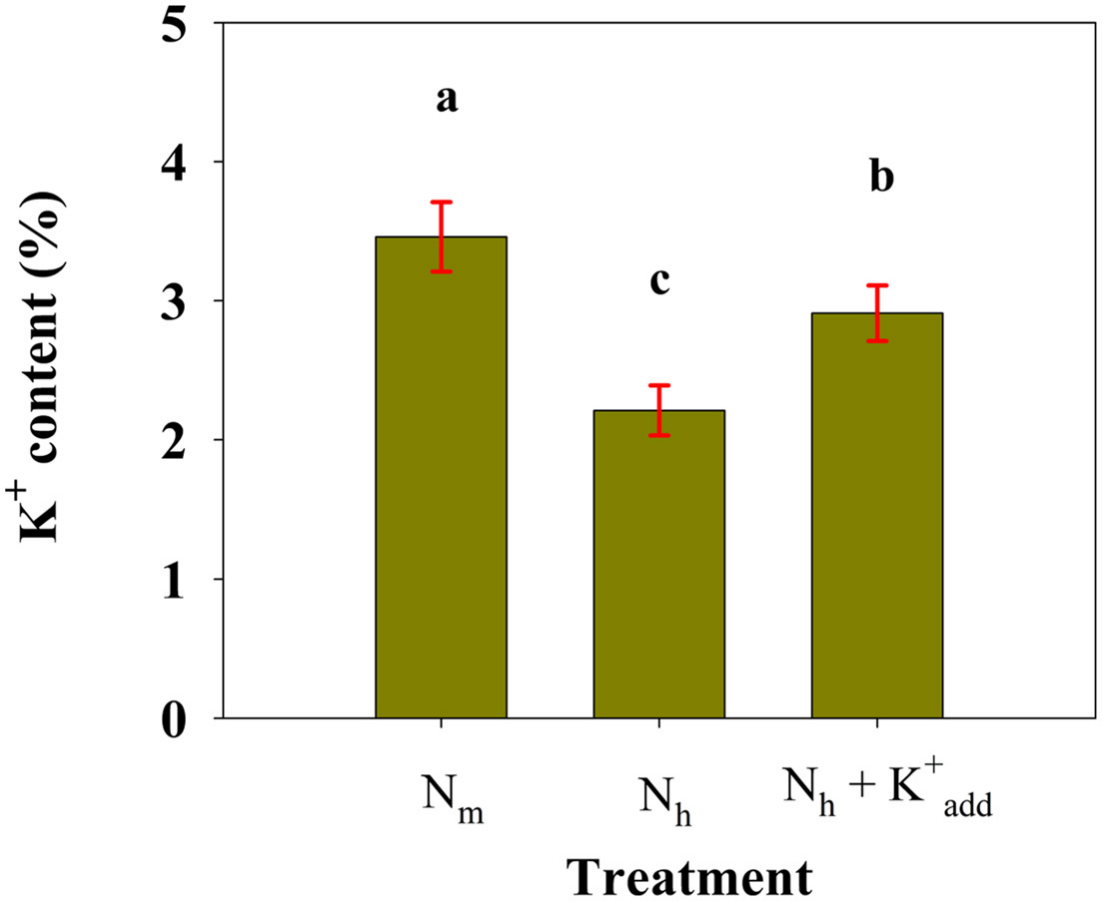
**Content of K^+^ (%) in 40-day-old wheat plants that were sand cultured in the presence of moderate NH^+^_4_ (full-strength Hoagland's nutrient solution; HNS) (N_m_), high NH^+^_4_ (full-strength HNS containing 10 mM NH_4_Cl) (N_h_) or high NH^+^_4_ combined with K^+^_add_ (HNS containing 10 mM NH_4_Cl and 6 mM KCl) (N_h_+K^+^_add_)**. The values are the means ± SD of five replicates. The different letters indicated above each column refer to significant differences at the *P* < 0.05 level.

**Figure 6:**
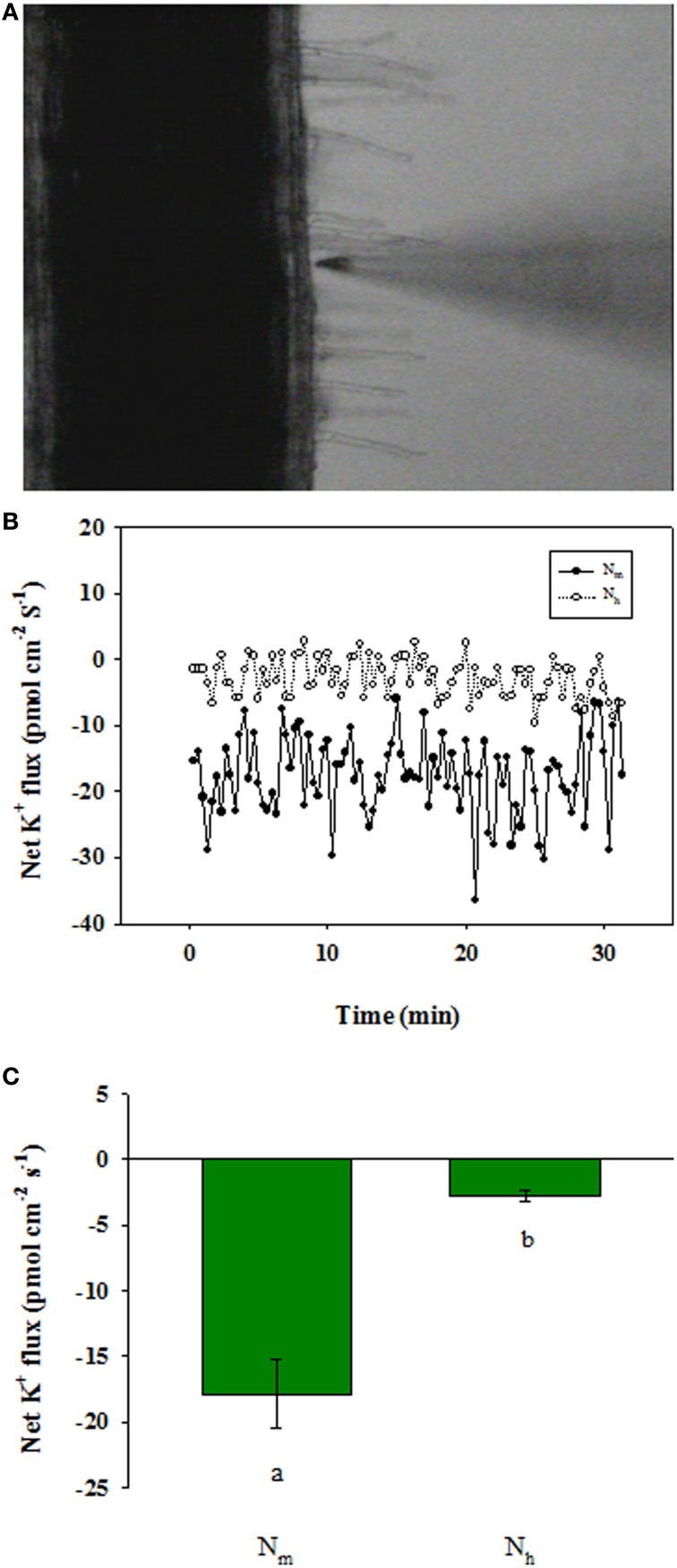
**Effects of high NH^+^_4_ (10 mM) on the kinetics of net K^+^ fluxes in the root epidermal cells of 15-day-old wheat seedlings. (A)** Wheat root tissue and the position of the electrode. K^+^ kinetics were recorded at a site approximately 1400 μm from the root tip). **(B)** The effects of 10 mM NH^+^_4_ on the kinetics of the net K^+^ fluxes in wheat root epidermal cells; each point is the mean from six individual plants. **(C)** Each column presents the mean values of the K^+^ fluxes measured in the root epidermal cells over a recording period of 30 min. N_m_: moderate NH^+^_4_, N_h_: 10 mM NH^+^_4_ treatment. The bars represent the standard deviation for six individual plants. The different letters indicated above each column refer to significant differences at the *P* < 0.05 level.

In the present study, a high NH^+^_4_ supply decreased the K^+^ content in wheat seedlings (Figure 5), indicating that high NH^+^_4_ suppressed K^+^ uptake by the plants. Furthermore, we examined the net K^+^ flux across the root epidermal cells in SIET experiments and further confirmed that high NH^+^_4_ decreased the K^+^ influx into the root epidermal cells (Figure 6). The results presented herein strongly suggest that uptake competition for NH^+^_4_ over K^+^ mediates K^+^ transport under high NH^+^_4_ conditions. This speculation is strongly supported by studies using intact barley seedlings, in which K^+^ fluxes into the root were shown to be much lower in seedlings grown using 10 mM NH^+^_4_ compared with seedlings grown using 10 mM nitrate (NO^−^_3_), and elevated K^+^ was able to ameliorate NH^+^_4_ toxicity (Kronzucker et al., 2003; Szczerba et al., 2006). The authors proposed that this protection may be associated with the restoration of a moderate K^+^ status to the plant, a process that ultimately depends on K^+^ fluxes into the roots and its subsequent translocation to the shoots (Kronzucker et al., 2003; Szczerba et al., 2006). The suppression of the K^+^ influx at the plasma membrane may be due to the inhibitory action of high NH^+^_4_ on high-affinity KUP/HAK/KT transporters (Spalding et al., 1999).

K^+^ and NH^+^_4_ exhibit numerous similarities, including their size and charge, and are poorly distinguished by some channels and transporters (Wang et al., 1996; White, 1996; Szczerba et al., 2008; Ariz et al., 2011). One hypothesis to explain how K^+^ ameliorates the toxicity of NH^+^_4_ is that K^+^ decreases the uptake of NH^+^_4_. Indeed, suppression of the NH^+^_4_ influx by K^+^ within minutes of increasing the K^+^ supply was observed in rice cultured in climate-controlled growth chambers under fluorescent lights (Balkos et al., 2010). Therefore, we examined this effect in wheat seedlings through SIET analyses and found that a high K^+^ rate did not alter NH^+^_4_ flux but stimulated NO^−^_3_ influx (data not shown) and thus high K^+^ treatment increased the total N uptake. This effect was also revealed by higher shoot N concentration in wheat plants under high K^+^ (Table 1). In field, plants grown under a slightly high NH^+^_4_ or NO^−^_3_ conditions show no toxicity on canopy growth but usually develop a weak culm mechanical strength as indicated by the higher lodging when strong wind occurs during grain filling. Given that the high K^+^ do not suppress the N uptake, the positive effects of K^+^ on cellulose and lignin deposition might be contributed to the additional K^+^ treatment in this study. Inhibition of K^+^ uptake by NH^+^_4_ has also been observed in *Arabidopsis* by Cao et al. (1993); these authors reported that the protective effect of K^+^ was not due to inhibition of NH^+^_4_ uptake. Additionally, as an intermediate of N metabolism and the most abundant amino acid in plants grown on NH^+^_4_-containing media (Hachiya et al., 2012), glutamine may be involved in the NH^+^_4_ toxicity. Therefore, experiments are required to investigate the relationships among glutamine content and K^+^ uptake and there effects on cellulose and lignin deposition and culm mechanical strength under different levels of K^+^ supply and to elucidate the mechanisms of the morphogenesis of cellulose and lignin phenotypes.

In summary, based on the data provided herein, we conclude that at high external concentrations, NH^+^_4_ decreases culm mechanical strength, cellulose and lignin contents, N remobilization from vegetative organs to the grain, the grain-filling rate and K^+^ uptake by wheat plants. These effects can be partially reversed by providing an additional K^+^ supply, most likely via competition with NH^+^_4_ uptake and translocation. Thus, an understanding of the roles of interaction between NH^+^_4_ and K^+^ in the regulation of culm mechanical strength and grain filling will be necessary to improve lodging resistance and productivity in wheat.

### Conflict of interest statement

The authors declare that the research was conducted in the absence of any commercial or financial relationships that could be construed as a potential conflict of interest.

## Supplementary material

Figure S1 **Forty-day-old sand-cultured wheat seedlings showing differences in culm mechanical strength under moderate NH^+^_4_ (full-strength HNS; (A), high NH^+^_4_ (full-strength HNS + 10 mM NH^+^_4_, (B) and high NH^+^_4_ with 6 mM additional K^+^ (C)**. The image represents five replicates.
