# Supplementary figures and images for: Effects of high NH+4 on K+ uptake, culm mechanical strength and grain filling in wheat
Source: Front Plant Sci. 2014 Dec 16;5:703. doi: 10.3389/fpls.2014.00703 (PMC4267191; doi:10.3389/fpls.2014.00703)

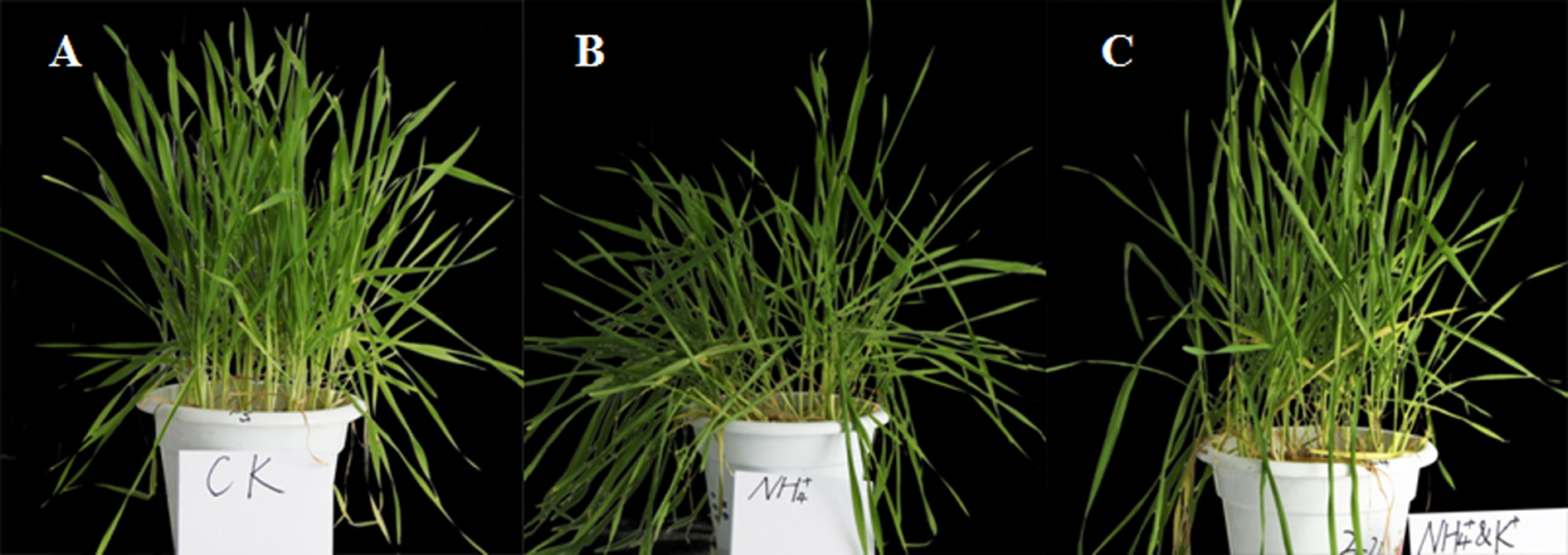

Supplement: Figure S1 — Forty-day-old sand-cultured wheat seedlings showing differences in culm mechanical strength under moderate NH+4 (full-strength HNS; (A), high NH+4 (full-strength HNS + 10 mM NH+4, (B) and high NH+4 with 6 mM additional K+ (C). The image represents five replicates. [file Image1.TIFF]
